# Economic burden of multiple sclerosis in Slovakia — from 2015 to 2020

**DOI:** 10.1186/s12913-022-08883-6

**Published:** 2022-12-02

**Authors:** Robert Babela, Judit Dugas

**Affiliations:** 1 Biomedical Research Center of the Slovak Academy of Sciences, Bratislava, Slovakia; 2 Institute for Healthcare Disciplines, St. Elizabeth University, Bratislava, Slovakia; 3 Slovak Medical University, Bratislava, Slovakia

**Keywords:** Cost-of-illness, Health care, Productivity loss, Multiple sclerosis

## Abstract

**Background:**

Multiple sclerosis (MS) is a chronic, inflammatory disease of the central nervous system, commonly diagnosed during young adulthood. The proportion of direct and indirect costs of MS vary across settings. The International Multiple Sclerosis Study, involving 1152 patients with MS from 19 countries, reported the average annual costs per patient to be €41,212, with direct medical costs of €21,093, direct non-medical costs of €2110, and €16,318 marked as indirect costs. However, there are no precise data on the economic burden of MS in Slovakia. Therefore, the main objective of this study is to assess the economic impact of MS in Slovakia by identifying and measuring the direct medical costs and indirect costs of this disease.

**Methods:**

We conducted a retrospective prevalence-based cost-of-illness analysis for MS in Slovakia sourced from the third-party payer and societal perspective. Patient co-payments and out-of-pocket expenses were not included in our study. We analysed all available costs and healthcare resources utilised in a 6-years period, from 2015 to 2020. For each year, all costs (in euro) were specified as total and the average annual cost per patient.

**Results:**

The estimated total economic burden of MS in Slovakia in 2020 was €57,347,523, with direct medical costs estimated to be €53,348,337 and indirect costs standing at €3,999,186. The total annual cost per patient in 2020 was €6682. Over the 6 years, the total diagnostic and treatment cost of patients with MS was estimated to be €283,974,236. With an average year-by-year increase of 5%, the total direct costs of MS had significantly grown during the examined 6 years. The total cost due to the MS-associated loss of productivity in these 6 years was €16,633,798. The average year-by-year increase of indirect costs of MS was 20%.

**Conclusions:**

Our study revealed the substantial health and economic burden of MS, with the average annual cost per patient to be approximately €6,682 in 2020. We provide the first extensive assessment of the burden of MS on Slovakian patients, the healthcare system, and society. It indicates the need for a detailed analysis of the employment of patients with MS to assess disability and work performance and the development of allied health policies.

**Supplementary Information:**

The online version contains supplementary material available at 10.1186/s12913-022-08883-6.

## Background

Multiple sclerosis (MS) is a chronic, inflammatory disease of the central nervous system, commonly diagnosed during young adulthood [1]. Signs and symptoms of MS may noticeably differ and are determined by the location and type of affected nerves. Common symptoms include numbness, walking and balancing difficulties, lack of coordination, vision problems, fatigue, problems with sexual, bowel and bladder function, and depression [1, 2]. Due to a broad spectrum of symptoms and its progressive character, MS profoundly impacts health-related quality of life. In most cases, it also leads to substantial disability [3–5].

Globally, females are twice as likely to develop the disease than males [6, 7]. The prevalence of MS has increased worldwide since 2013. In 2020, about 2.8 million people (35.9 per 100,000 population) were estimated to be living with MS worldwide, which was ⁓30% higher than in 2013 [7]. The prevalence and incidence of MS vary across the geographical region, with the highest prevalence observed in Europe and America while the lowest in Africa, South East Asia, and Western Pacific regions [8]. The 2020 MS Barometer, including data from 35 European countries, estimated about 1,188,000 people with MS in Europe [9]. The prevalence was found to be the highest in Germany (299 per 100,000 people), Denmark (289 per 100,000 people) and Norway (240 per 100,000 people), and the lowest in Moldova (37 per 100,000), Belarus (40 per 100,000 people), and Romania (47 per 100,000 people) [9].

Since MS is typically diagnosed between 20 and 50 years of age, it affects education, career, and family life, with negative consequences on patients and their caregivers’ working activity [7, 10]. A significant proportion of patients with MS declare unemployment or reduced work participation, mainly due to the increasing severity of the disease and resulting impairments [11]. Unemployment rates in this population are as high as 60–80% [12, 13]. Due to productivity loss and the long-term need for specialist treatment, MS places a substantial economic burden on healthcare systems and societies [14]. In the early stages of the disease, costs of illness are mainly driven by direct medical costs [15]. Along with the increased severity, the overall costs rise steadily and substantially. Similarly, the indirect costs also escalate caused by reduced work productivity due to short- or long-term sick leave, disability pension, early retirement, or premature death.

The proportion of direct and indirect costs of MS vary across settings. Encompassing 1152 MS patients from 19 countries, the International Multiple Sclerosis Study stated the average annual costs for each patient to be €41,212, with direct medical costs standing at €21,093, direct non-medical costs of €2110, and indirect costs to be €16,318 [16]. A cross-sectional study from 16 European countries presented the total mean annual costs per patient with MS to be € 22,800 for mild disease, € 37,100 for moderate disease, and € 57,500 for severe MS [17]. Indirect costs account for a significant part of the total costs of MS and vary depending on the country, from 12 to 54% [18–31].

So far, limited information on the prevalence, incidence, and cost of MS in Slovakia has been published. Some estimations were made in the Atlas of Multiple Sclerosis in 2008 and by GBD 2016 Multiple Sclerosis Collaborators [32, 33]. We also found one cost-of-illness study evaluating the economic impact of MS in Slovakia by Pšenková and colleagues. The total annual costs in 2010 estimated by the authors were €54,723,592, with indirect costs of €31,728,757 and the average cost per patient being €8971 [34]. This analysis, however, only collected data from one insurance company.

Evidence of MS costs estimated from payers and societal perspectives are available for most European countries, but no current data for Slovakia has been published so far. Therefore, we aim to assess the economic impact of MS in Slovakia by identifying and measuring the direct medical costs and indirect costs of this disease.

## Methods

### Analysis framework and data sources

This retrospective prevalence-based cost-of-illness analysis for MS in Slovakia was conducted based on the third-party payer and societal perspective. From the third-party payer perspective, the analysis included all direct medical costs paid by the insurance companies. The analysis from the societal perspective also considered the costs of decreased or lost productivity of the patient with MS. Patient co-payments and out-of-pocket expenses were not included in the study. We analysed all costs and healthcare resources used in a 6-years period, from 2015 to 2020. All costs were expressed as total and the average annual cost per patient in a respective year. All estimated costs were expressed in euro. No discounting of costs was applied.

MS was defined according to the WHO ICD-10 classification as ICD code G35. Patients were divided based on disease severity. Data on the prevalence of MS in Slovakia over 2015–2020 were obtained from the National Health Information Center (http://www.nczisk.sk/Statisticke_vystupy/Tematicke_statisticke_vystupy/Neurologia/Pages/default.aspx). Information on the age-sex structure of the population, and the subdiagnoses were obtained from Institute for Health Analysis (https://www.health.gov.sk/?iza).

We carried out a detailed analysis of reimbursement expenditure between 2015 and 2020, based on the reimbursement data published by the following sources:

- General Health Insurance Company (Vseobecna zdravotna poistovna; VsZP; www.vszp.sk).
- Privately-owned Dovera health insurance company (www.dovera.sk).
- Union health insurance company (www.union.sk).
- The Ministry of Health.
- Institute of Health Analysis (www.health.gov.sk).

The available data sets also included the cost of outpatient diagnostic and medical procedures.

While calculating the indirect costs generated by patients with MS in Slovakia between 2015 and 2020, we analysed the aggregated data on days spent on paid sick leave from work due to MS and the number of patients with disability due to MS provided by the Social Insurance Agency (www.socpoist.sk) for the selected period.

### Costs

In our analysis, we considered both direct medical costs and indirect costs generated by patients with MS. Direct medical costs included: (i) inpatient care costs (all hospital visits longer than 48 h with the MS ICD-10 code G35.XX as the primary diagnosis, procedures reimbursed on top of the hospitalization package fee, reimbursed spa stay, and others), (ii) pharmacotherapy, (iii) diagnostic and medical procedures. Costs of medical treatment included all drugs reimbursed for patients with MS in Slovakia separately or together with hospitalization. Costs of outpatient diagnostic and medical procedures included: initial and differential MRI examinations, cerebrospinal fluid examination, visual evoked potentials, PET scans, complete biochemical blood or cerebrospinal fluid tests, differential diagnostic tests, and rehabilitation costs. Our analysis has not assessed direct non-medical costs, e.g., formal care in homes and care homes or informal care, due to the lack of such data in Slovakia. We excluded premature mortality costs due to relatively small and difficult to assess impact of MS on mortality.

Indirect costs included in the analysis were: costs of short-term absence from work, long-term absence from work, and early retirement. Productivity loss due to absenteeism was estimated as the number of days spent on paid sick leave from work due to G35.XX diagnosis multiplied by the national average salary in the years 2015–2020 (Additional file 1).

The disability costs were calculated as a proportion of the total number of people with G35.XX who were granted an invalidity pension multiplied by lump sum benefit provided by the Slovakian Social Insurance Agency for the disability (Additional file 2).

### Data analysis

We performed the retrospective analysis using the anonymised electronic health-insurance patients’ data. We worked with data from all health insurance companies in Slovakia that jointly pay for the healthcare services for the entire population. A major part of the data was extracted from the state-owned health insurance company Všeobecná zdravotná poisťovňa [35]. The direct costs data were collected for 6 consecutive years from 2015 to 2020. The data represents every insured person who had any reimbursed care for the diagnosis of MS (International Classification of Diseases, 10th revision, ICD-Code: G35, including all the sub-diagnosis codes G35.0-G35.9). Patients received either outpatient or inpatient care, or both.

Out-of-pocket costs associated with drug co-payments, travel to the hospital or the specialist’s office, and taking care of the sick relative were omitted since no database would collect these data. Furthermore, there is no current published research that would collect out-of-pocket expenses on a representative sample of patients with MS in Slovakia. Owners of the datasets rely on the correctness of the diagnosis information and related costs from the HCP’s monthly claims. Specialists’ visits were included in the reimbursed care as well.

For indirect costs, data associated with absenteeism and disability were provided by the Social Insurance Agency The data was divided into two major parts: costs associated with absenteeism due to illness and disability pensions that were verified by the authority in each selected year. Additionally, datasets included average absence from work in days from 2015 to 2020.

## Results

### Population

According to epidemiologic data from the National Health Information Centre, there were 6762 prevalent cases of MS in Slovakia in 2015 (Table 1). The prevalence of MS rose remarkably over the years and reached 8582 cases in 2020 (a 27% increase compared to 2015).

**Table 1:** Overall estimated prevalence of MS (ICD-10: G35; 2015–2020 year)

	2015	2016	2017	2018	2019	2020
**All Patients**	6762	5048	7483	7790	8468	8582

On average, 68.9% of the patients were female, and about 97.2% were below retirement age (Table 2). In the study population, 58.1% of patients were diagnosed as ICD-10 code G35.10 (MS with a relapsing-remitting course, without signs of relapse or progression), 27.9% were diagnosed as G35.11 (MS with a relapsing-remitting course, with signs of relapse or progression), 5.1% as G35.0 (MS, first manifestation), and 4.4% as G35.9 (MS, not specified) (Table 3). 

**Table 2:** Proportions of patients with MS according to sex and age categories (2015–2020 year)

	2015	2016	2017	2018	2019	2020
**age, %**						
** 0–17**	0.71	0.38	0.41	0.33	0.36	0.37
** 18–64**	96.61	97.53	97.37	97.64	95.33	96.03
** 65+**	2.68	2.08	2.22	2.03	4.31	3.60
**sex, %**						
** Male**	29.8	30.5	30.7	30.7	32.0	32.0
** Female**	70.2	69.5	69.3	69.3	68.0	68.0

**Table 3:** Proportions of patients according to most common sub-diagnose categories of MS (2015–2020 year)

	2015	2016	2017	2018	2019	2020
	**%**					
**G35.10**	52.4	60.8	50.3	53.2	62.0	68.3
**G35.11**	21.7	23.4	37.0	36.6	24.0	20.0
**G35.9**	9.8	5.3	4.8	3.0	3.6	2.8
**G35.0**	8.6	6.0	4.5	4.0	5.3	4.1
**Others**	7.5	4.5	3.4	3.2	5.1	4.8

### Total direct and indirect costs

The estimated total economic burden of MS in Slovakia in 2020 was €57,347,523, with direct medical costs estimated to be €53,348,337 and reduced productivity costs being €3,999,186 (Table 4). The total annual cost per patient in 2020 was €6682 (Table 5).

**Table 4:** Total costs of MS (ICD-10: G 35; 2015–2020 year)

Cost group/Year	2015	2016	2017	2018	2019	2020
**Hospitalisation, €**	1 563 398	1 714 724	1 762 691	1 985 472	2 236 369	2 112 815
**Pharmacotherapy, €**	37 629 952	38 465 909	41 247 081	43 876 949	47 628 108	48 476 067
**Diagnostics and special therapeutic/medical procedures, €**	2 411 447	2 410 214	2 431 565	2 592 783	2 669 237	2 759 455
**Total direct costs, €**	**41 604 798**	**42 590 847**	**45 441 337**	**48 455 204**	**52 533 714**	**53 348 337**
**Absenteeism (sickness benefits)**	1 047 144	1 020 946	1 119 886	1 160 903	1 238 580	1 260 604
**Disability (invalidity/long term disability)**	596 119	976 385	1 382 586	1 762 157	2 329 906	2 738 582
**Total indirect costs**^a^, €	**1 643 263**	**1 997 331**	**2 502 472**	**2 923 060**	**3 568 486**	**3 999 186**
**Total costs of MS, €**	**43 248 061**	**44 588 178**	**47 943 809**	**51 378 263**	**56 102 200**	**57 347 523**

^a^sum of absenteeism cost (sickness benefits) and disability cost (invalidity/long term disability)

**Table 5:** Direct medical, indirect, and total costs per patient of MS (ICD-10: G 35; 2015–2020 year)

Cost group/Year	2015	2016	2017	2018	2019	2020
**Total direct costs per patient, €**	6153	8437	6073	6220	6204	6216
**Total indirect costs per patient, €**	243	396	334	375	421	466
**Total costs of MS per patient, €**	6396	8833	6407	6595	6624	6682

Over the 6 years, the total diagnostic and treatment cost of patients with MS were estimated to be €283,974,236. The total direct costs of MS increased significantly during the investigated 6 years. In 2020, the medical cost of MS was €53,348,337 resulting in €6216 annual cost per patient and was 28% (€11,743,539) higher than in 2015. The average year-by-year increase in the direct cost of MS was 5%. The major components of medical expenditures were medicines that remined about 91% of direct costs each year. At the same time, costs associated with hospitalisation, diagnostic and medical procedures constituted about 9% of medical costs. The total costs of loss of productivity loss due to MS over the 6 years were €16,633,798. These costs increased significantly during the explored 6 years. In 2020, the cost of productivity loss due to MS was €3,999,186, resulting in €466 annual cost per patient, which was 143% (€2,355,923) higher than in 2015. The average year-by-year increase of indirect costs of MS was 20%. The key driver of costs of MS were direct costs. In 2020, the estimated costs of medical care remained at about 93% of the total costs of MS. The proportion of direct costs in total costs was constant during the analysed years and accounted for about 95%.

## Discussion

This study is the first prevalence-based analysis estimating the economic burden of MS in Slovakia. Using the societal perspectives, we have estimated that the annual costs of MS in 2020 in Slovakia were approximately €57.3 million. Costs of medical treatment and productivity loss due to absenteeism and disability represented 93% (€53.3 million) and 7% (€4.0 million) of the total costs, respectively. The main cost driver was the pharmacotherapy of patients with MS, corresponding to €48.5 million (85% of total cost).

As it has been mentioned before there is inadequate amount of published data on MS prevalence in Slovakia. Moreover, published estimates are not up to date and are inconsistent. Estimations provided in the Atlas of Multiple Sclerosis, a world compendium of data on MS epidemiology, showed a yearly incidence of 7.5 per 100 000 people and 8,500 people living with MS in 2008 in Slovakia [32]. The GBD Multiple Sclerosis Collaborators reported 3,372 patients with MS in 2016, with a 33.5% increase in morbidity between 1990 and 2016 [33]. Our investigation reveals the current epidemiological data based on the estimations gathered by the National Health Information Centre. According to these aggregated data, the number of patients treated with MS in Slovakia was rising year by year and accounted for 6,762 in 2015 and 8,582 in 2020. It is generally in line with the prevalence of MS observed over recent years worldwide. The estimates presented in the Atlas of Multiple Sclerosis indicated an increasing prevalence of MS in all WHO regions, with a 30% higher number of cases in 2020 compared to 2013 [32]. A similar increase (27%) was observed in our study for a comparable timeframe (from 2015 to 2020). This upward trend corresponds to increased life expectancy and decreased mortality of patients with MS.

Previous population-based studies focusing on different geographic regions indicated that females are twice as likely to have MS as males [6, 7]. This relationship is also evident in Slovakian patients with MS, of which 68% are females. It has been noted in numerous epidemiological studies that MS commonly occurs between 20 and 50 years of age and affects people of productive age [36, 37]. Therefore, this illness has severe consequences on a person’s ability to remain in the workforce. It is clearly visible in the Slovakian population, where about 96% of patients are of productive age (18–64 years).

In the analyses of different European regions, Paz-Zulueta and colleagues estimated total annual costs per patient with MS from a societal perspective in 2015 to be €44,589 in Northern Europe (Denmark, Finland, Ireland, Sweden, and the United Kingdom), €47,619 in Western Europe (Austria, Belgium, France, Germany, the Netherlands, and Switzerland), €36,978 in Southern Europe (Italy, Portugal, and Spain) and €15,205 in Eastern Europe (the Czech Republic, Hungary, and Poland), with average total annual cost per patient in Europe being €40,303 [14]. Latest analysis from 2022 from Italy by Battaglia and colleagues confirmed that MS carries a substantial burden to patients and society with mean annual costs at €39,307 per patient [38]. Our analysis estimated that the mean total costs per patient with MS were €6,922 (years 2015–2020), about 2.5-fold lower than reported in cost-of-illness studies conducted previously for Eastern European countries and 5.7 to 6.5-fold lower than the average total annual cost per European patient with MS including recently published data from Italy [38].

The interpretation of MS cost differences between Slovakia and other European regions is challenging due to the lack of comprehensive, updated systemic data on Slovakian patients with MS. Nonetheless, one of the probable reasons for these differences could be the underestimation of the actual number of Slovakian patients with MS due to the lack of a national registry and published epidemiological surveys. The current analysis is based only on the published data from the National Health Information Centre. However, this aggregated data may not include patients during the diagnostic process or those misdiagnosed. Moreover, this analysis includes only formal state-granted disability and does not cover any informal expenses, costs of sequelae, and out-of-pocket payments. Thus, the underestimation relates also to disability costs and medical costs.

Most of the published data on MS costs demonstrate a significant impact of direct medical costs on total costs generated by patients with MS. This is in line with other studies for neurological disorders where medical costs represent a significant part of the total costs of illness [38–40]. In our analysis, these costs were about €6,216 in 2020 and accounted for 93% of total costs. Healthcare costs were, as expected, dominated by the cost of pharmacotherapy (which was responsible for 90% of medical costs incurred in 2020). In most published cost-of-illness studies, the proportion of indirect costs rose along with the deterioration of the patient’s disability [38, 41]. The main cost drivers for individuals with low disease severity are drugs (accounted for 29–82% of total costs). In contrast, for patients with more advanced MS symptoms, the cost of productivity loss due to MS is a significant factor (representing 17–67% of total costs) [15]. The significant share of pharmacotherapy costs in medical and total costs of MS in our analysis results from the fact that patients treated with disease-modifying drugs represent a substantial proportion of the Slovakian MS population. The previous estimations in the sole published study on the burden of MS in Slovakia by Pšenková and colleagues presented the outweighing of the indirect cost by the direct cost. Although, the proportion of cost components in that study differed significantly from our results, with indirect costs we carried out a detailed analysis figured at €31,729 and direct costs at €22,995. Nevertheless, the abovementioned analysis included data from only one insurance company and applied the calculation of indirect costs by the human capital approach method [34]. In the timeframe of our analysis, we noted a considerable increase in the total pharmaceutical expenditures from €37,629,952 in 2015 to €48,476,067 in 2020 (an increase of 29%). This change is mainly driven by an intensified use of the disease-modifying drugs due to a steady increase in MS prevalence and an increasing number of people living with MS resulting from decreased mortality due to MS.

MS causes immense indirect costs due to productivity loss. Comparing to other MS cost studies, we observed lower productivity loss costs resulting from sick leave and disability pension, ultimately contributing to lessening the total MS cost. These costs were nearly €4.0 million in 2020 — 2.5-fold higher than in 2015. As described by Paz-Zulueta and colleagues, indirect costs comprised about 23% (range 9–36%) of total MS costs [14]. Recent study by Battaglia and colleagues confirmed increased indirect costs burden with increased severity of the patient with overall 39% share (range 30–42%) from total MS costs in Italy in 2019 [38]. Cross-sectional study conducted in 16 countries by the MSCOI Study Group and the European Multiple Sclerosis Platform shows a wide variation of the contribution of indirect costs depending on the country and disease activity from 46% to even 82%, comprising on average approximately 27% in mild and 37% in mild and moderate disease. In our estimations, indirect costs consisted of only 7% of the total costs [33].

## Conclusions

In conclusion, our study revealed the substantial health and economic burden on Slovakian patients with MS. The increase in costs is evident from €43.2 million in 2015 to €57.3 million in 2020, with the average annual cost per patient estimated to be €6,682 in 2020. Pharmacotherapy was the main cost driver accounting for about 85% of the total cost (€48.5 million in 2020). This study provides the first extensive assessment of the burden of MS on Slovakian patients, the healthcare system, and society. It indicates the need for a detailed analysis of the employment of MS patients aiming at assessment of disability and work performance and the development of related health policies.

## Supplementary Information

**Additional file 1**. Average salary in industry in Slovakia (2015-2019 year).

**Additional file 2**. Disability lump sum rates in Slovakia (2015-2019 year).

## Data Availability

The datasets used and/or analysed during the current study are available and can be accessed from the corresponding author on reasonable request.

## Abbreviation

MS: Multiple sclerosis
