# Supplementary material for: Economic burden of multiple sclerosis in Slovakia — from 2015 to 2020
Source: BMC Health Serv Res. 2022 Dec 2;22:1467. doi: 10.1186/s12913-022-08883-6 (PMC9717442; doi:10.1186/s12913-022-08883-6)
Supplement: Supplementary file 1 — Additional file 1. Average salary in industry in Slovakia (2015-2019 year). [file 12913_2022_8883_MOESM1_ESM.docx]

Additional file 1 Average salary in industry in Slovakia (2015-2019 year).

| **Year** | **Value/ Unit value** | **Source** |
| --- | --- | --- |
| 2015 | €883 | https://www.finance.sk/mzda/priemerna-mzda/ |
| 2016 | €912 |  |
| 2017 | €954 |  |
| 2018 | €1,013 |  |
| 2019 | €1,092 |  |
| 2020 | €1,133 |  |
