# Supplementary material for: Economic burden of multiple sclerosis in Slovakia — from 2015 to 2020
Source: BMC Health Serv Res. 2022 Dec 2;22:1467. doi: 10.1186/s12913-022-08883-6 (PMC9717442; doi:10.1186/s12913-022-08883-6)
Supplement: Supplementary file 2 — Additional file 2. Disability lump sum rates in Slovakia (2015-2019 year). [file 12913_2022_8883_MOESM2_ESM.docx]

Additional file 2 Disability lump sum rates in Slovakia (2015-2019 year).

| **Year** | **Formally confirmed disability level** | | **Source** |
| --- | --- | --- | --- |
|  | **<70%** | **≥70%** |  |
| 2015 | €197.51 | €352.49 | https://www.socpoist.sk/priemerna-vyska-vyplacanych-dochodkov--v-mesiacoch-/1600s, access 31.12.2020 |
| 2016 | €197.04 | €353.18 |  |
| 2017 | €200.05 | €359.88 |  |
| 2018 | €203.69 | €368.03 |  |
| 2019 | €209.85 | €379.95 |  |
| 2020 | €217.79 | €394.74 |  |
